# *Ascophyllum nodosum* and *Fucus vesiculosus* Extracts Improved Lipid Metabolism and Inflammation in High-Energy Diet–Induced Hyperlipidemia Rats

**DOI:** 10.3390/nu14214665

**Published:** 2022-11-04

**Authors:** Yu-Tang Tung, Chieh-Hsi Wu, Wen-Chao Chen, Chun-Hsu Pan, Yi-Wen Chen, Shu-Ping Tsao, Chia-Jung Chen, Hui-Yu Huang

**Affiliations:** 1 Graduate Institute of Biotechnology, National Chung Hsing University, Taichung 402, Taiwan; 2 Graduate Institute of Metabolism and Obesity Sciences, Taipei Medical University, Taipei 110, Taiwan; 3 Nutrition Research Center, Taipei Medical University Hospital, Taipei 110, Taiwan; 4 Cell Physiology and Molecular Image Research Center, Wan Fang Hospital, Taipei Medical University, Taipei 116, Taiwan; 5 School of Pharmacy, Taipei Medical University, Taipei 110, Taiwan; 6 Ph.D. Program in Drug Discovery and Development Industry, College of Pharmacy, Taipei Medical University, Taipei 110, Taiwan; 7 Division of Gastroenterology and Hepatology, Department of Internal Medicine, Taipei Medical University Hospital, Taipei 110, Taiwan; 8 Research Center for Digestive Medicine, Taipei Medical University Hospital, Taipei 110, Taiwan; 9 Neuroscience Research Center, Taipei Medical University, Taipei 110, Taiwan

**Keywords:** phlorotannins, high-energy diet, hyperlipidemia, *Ascophyllum nodosum*, *Fucus vesiculosus*

## Abstract

*Ascophyllum nodosum* and *Fucus vesiculosus* both contain unique polyphenols called phlorotannins. Phlorotannins reportedly possess various pharmacological activities. A previous study reported that the activity of phlorotannin is strongly correlated with the normalization of metabolic function, and phlorotannins are extremely promising nutrients for use in the treatment of metabolic syndrome. To date, no study has explored the antihyperlipidemic effects of phlorotannins from *A. nodosum* and *F. vesiculosus* in animal models. Therefore, in the present study, we investigated the effects of phlorotannins using a rat model of high-energy diet (HED)-induced hyperlipidemia. The results showed that the rats that were fed an HED and treated with phlorotannin-rich extract from *A. nodosum* and *F. vesiculosus* had significantly lower serum fasting blood sugar (FBS), aspartate aminotransferase (AST), alanine aminotransferase (ALT), total cholesterol (TC), triacylglyceride (TG) and free fatty acids (FFAs) levels and hepatic TG level and had higher serum insulin, high-density lipoprotein cholesterol (HDL-C) levels and lipase activity in their fat tissues than in the case with the rats that were fed the HED alone. A histopathological analysis revealed that phlorotannin-rich extract could significantly reduce the size of adipocytes around the epididymis. In addition, the rats treated with phlorotannin-rich extract had significantly lowered interleukin 6 (IL-6) and tumor necrosis factor alpha (TNF-α) levels and increased superoxide dismutase (SOD) and glutathione peroxidase (GPX) activities than did those in the HED group. These results suggested that the phlorotannin-rich extract stimulated lipid metabolism and may have promoted lipase activity in rats with HED-induced hyperlipidemia. Our results indicated that *A. nodosum* and *F. vesiculosus*, marine algae typically used as health foods, have strong antihyperlipidemic effects and may, therefore, be useful for preventing atherosclerosis. These algae may be incorporated into antihyperlipidemia pharmaceuticals and functional foods.

## 1. Introduction

Atherosclerosis is a chronic inflammatory response of the walls of the arteries and is the leading cause of heart disease, stroke, and death worldwide. Elevated serum total cholesterol (TC) and triglyceride (TG) levels are primary risk factors for atherosclerosis [1]. Recently, research on lowering or regulating serum TC and TG levels has become more common. Researchers have reported that dietary plants with cholesterol-lowering activity may be useful for preventing atherosclerosis [2,3,4].

Marine algae are widely distributed and abundant in the coastal areas of many countries [5], and their secondary metabolites are sources of natural compounds with beneficial pharmaceutical properties. Several researchers have explored the pharmacological effects of polyphenolic compounds from marine algae [6,7]. In Asian countries, brown algae have long been consumed as food items and folk medicines. *Ascophyllum nodosum* and *Fucus vesiculosus* are brown algae that contain unique polyphenols called phlorotannins [8]. Phlorotannins possess antioxidant [9], anti-inflammatory [10], antibacterial [11], anti-HIV [12], enzyme-inhibitory (against α-glucosidase, α-amylase, acetylcholinesterase, butyl cholinesterase, angiotensin-I-converting enzyme, matrix metalloproteinases, hyaluronidase, and tyrosinase) [13], and anticarcinogenic [14] properties.

Phlorotannins from some brown algae inhibit α-amylase and α-glucosidase, which are involved in carbohydrate digestion and assimilation [15]. Phlorotannins have demonstrated multiple anti-diabetic mechanisms such as α-amylase and α-glucosidase inhibition, glucose uptake in skeletal muscle, protein tyrosine phosphatase 1B (PTP 1B) enzyme inhibition, improvement of insulin sensitivity in type 2 diabetic db/db mice [16]. In addition, the phlorotannin-rich extracts of *Ecklonia stolonifera* suppress the increase in plasma glucose and lipid peroxidation levels in male KK-Ay mice in a mouse model of genetically non-insulin-dependent diabetes [17]. A high molecular weight phlorotannins from *Sargassum thunbergii* could significantly decrease TC, TG, and LDL-C levels, mainly dependent on the elevation of hepatic LDL-R levels [18]. Phlorotannins from *A. nodosum* and *F. vesiculosus* reduce the normal increase in postprandial blood glucose 30 min after a meal by 90% and decrease peak insulin secretion by 40% [8]. Eckol and dieckol, phlorotannins from *E. stolonifera*, significantly reduce serum TG and TC levels and atherogenic index values in a rat model [19].

Previous studies have indicated that phlorotannin activity is strongly correlated with the normalization of metabolic function. Therefore, phlorotannins are considered promising nutrients for use in the treatment of metabolic syndrome. To date, no study has explored the antihyperlipidemic effects of phlorotannins from *A. nodosum* and *F. vesiculosus*. In this study, we investigated the effects of phlorotannins using a rat model of high-energy diet (HED)-induced hyperlipidemia.

## 2. Materials and Methods

### 2.1. Extraction

*A. nodosum* and *F. vesiculosu* was extracted in hot water. The extracts were subjected to a cycle of filtration and ultrafiltration and ultimately spray dried. The brown algae crude extract used in this study was supplied by LICA Enterprises (Taipei, Taiwan) and is available commercially under the tradename InSea2.

### 2.2. Determination of Phlorotannins

The total phenolic contents were determined according to the Folin-Ciocalteu method. In this case, 0.5 mL of extract solution was allowed to react with 0.5 mL of 1 N Folin-Ciocalteu reagent. After 2–5 min, 1.0 mL of 20% Na_2_CO_3_ was added. The mixture was kept within 10 min, then centrifuge for 8 min (12,000× *g*). The absorbance was recorded at 730 nm. The results were expressed as gallic acid equivalents (GAE) in milligrams per gram of extracts.

### 2.3. Animals

Seven-week-old male Sprague Dawley rats were provided by BioLasco (Taipei, Taiwan). The rats were housed at a relative humidity of 50 ± 10% and a temperature of 22 ± 2 °C with a 12-h (07:00–19:00) light/dark cycle throughout the experiment. The rats were provided water and food ad libitum throughout the experiment. After a 1-week acclimatization period, the rats were provided a normal diet (ND; n = 10; Laboratory Rodent Diet 5001, LabDiet, St. Louis, MO, USA; 13% fat with an energy density of 2.89 kcal/g) or an HED (n = 40; Diet #D12468, Research Diets, New Brunswick, NJ, USA; 47.7% fat with an energy density of 4.81 kcal/g) for 4 weeks. Thereafter, the HED-fed rats were divided into four experimental groups (n = 10/group) and were untreated (HED group) or were treated with a low dose (25.83 mg/kg; HED/LCE group), medium dose (51.66 mg/kg; HED/MCE group), or high dose (103.33 mg/kg; HED/HCE group) of brown algae crude extract. The brown algae crude extract was dissolved in distilled water and administered using an oral gavage once daily for 8 weeks, while continuing the HED. The ND and HED groups received distilled water alone. Each rat’s body weight, water intake, and diet consumption were measured was measured once per week throughout the study. The rats were fasted for 12 h after the final treatment and anesthetized; the blood samples were collected through cardiac puncture with nonheparinized syringes and incubated on ice for 30 min. The blood samples were centrifuged at 1500× *g* for 10 min at 4 °C for serum separation. The rats’ livers, epididymal fat, perirenal fat, mesenteric fat, and brown adipose tissue were immediately excised, rinsed, weighed, snap-frozen in liquid nitrogen, and stored at −80 °C until subsequent analyses. In addition, the fat around the epididymis was fixed in 4% formaldehyde for histopathological examination. The study protocol was approved by the Institutional Animal Care and Use Committee of Taipei Medical University (LAC-2019-0417), and the study was performed in accordance with the Guide for the Care and Use of Laboratory Animals published by the National Institutes of Health (NIH).

### 2.4. Serum Biochemical Parameter Measurement

The rats’ serum fasting blood sugar (FBS), insulin, aspartate aminotransferase (AST), and alanine aminotransferase (ALT), uric acid, creatinine, Na^+^, K^+^, and ketone body levels were measured using a Roche Modular P800 (Roche Diagnostics, Indianapolis, IN, USA).

### 2.5. Measurement of Lipid Index

The rats’ serum levels of lipid indices, including TC, TG, high-density lipoprotein cholesterol (HDL-C), low-density lipoprotein cholesterol (LDL-C) and free fatty acids (FFAs) levels, were measured using a Roche Modular P800 (Roche Diagnostics, Indianapolis, IN, USA). Each liver tissue sample was homogenized, and the homogenate was centrifuged at 10,000× *g* for 10 min at 4 °C. The supernatant was stored on ice for hepatic TG and TC assays. Hepatic TC and TG were measured using an enzyme-linked immunosorbent assay colorimetric assay kit including TC colorimetric assay kit (Elabscience, Houston, TX, USA; Cat. No. E-BC-K109-M) and a TG colorimetric assay kit (Elabscience, Houston, TX, USA; Cat. No. E-BC-K238), respectively.

### 2.6. Serum Proinflammatory Factor and Antioxidant Enzyme Activity Level Measurement

The serum levels of proinflammatory factors were measured using sandwich ELISA kits for interlukin-6 (IL-6; Elabscience, Houston, TX, USA; Cat. No. E-EL-R0015) and tumor necrosis factor-α (TNF-α; Elabscience, Houston, TX, USA; Cat. No. E-EL-R0019). The antioxidant enzyme activities were determined using sandwich ELISA kits for superoxide dismutase (SOD; Elabscience, Houston, TX, USA; Cat. No. E-BC-K022-M) and glutathione peroxidase (GPx; Elabscience, Houston, TX, USA; Cat. No. E-BC-K096-M).

### 2.7. Measurement of Lipase Activity in the Adipose Tissue

In the lipase activity measurement process, the epididymal fat pad was homogenized and centrifuged using a cold microcentrifuge at top speed for 2–5 min at 4 °C to remove any insoluble material. The supernatant was measured using a Lipase Assay Kit (colorimetric; CAT# ab102524, Abcam Inc., Cambridge, MA, USA) in accordance with the manufacturer’s specifications.

### 2.8. Mitochondrial Function Assessment

Mitochondrial function was assessed according to the rats’ real-time basal oxygen consumption rates (OCRs), which were measured using a Seahorse Bioscience XFe24 Extracellular Flux Analyzer. After the rats were sacrificed, samples of their subcutaneous fat were transferred into an XFe24 islet capture microplate (Seahorse Bioscience, Billerica, MA, United States, Cat. No. 101122-100). The samples were analyzed using a method described in a previous study [20].

### 2.9. Histopathological Analysis

After the rats were sacrificed, samples of their epididymal white adipose tissue (eWAT) were dissected, collected, and fixed in 4% formaldehyde. The samples were processed in a graded series of alcohols and embedded in paraffin wax, divided into 4-μm-thick sections, stained with hematoxylin and eosin for histological examination, and imaged at 10X magnification. A clinical pathologist examined the sections under an optical microscope (Olympus, Shinjuku-ku, Tokyo, Japan). In this case, 100 adipocytes were randomly sampled from each section using the manual function of the system, and the distribution areas of individual adipocytes were recorded. The adipocyte areas were analyzed using the ImageJ software package (NIH, Bethesda, MD, USA).

### 2.10. Fecal Microbiota Analysis

The day after the last supplementation, fresh fecal samples were collected individually from each rat through abdominal massage. The DNA was isolated using a QIAamp Fast DNA Stool Mini Kit (Qiagen, Germantown, MD, USA) according to the manufacturer’s instructions. The V3–V4 region of the bacterial 16s rRNA gene was amplified using a PCR method. Thereafter, a Nextera XT Index Kit (Illumina, San Diego, CA, USA) was used to label the amplicons. After the amplicons were evaluated for amplification product quality using a Fragment Analyzer (Advanced Analytical, Ankeny, IA, USA), the amplicons were quantified using a Qubit dsDNA HS assay kit (Life Technologies, Pleasanton, CA, USA), according to the manufacturer’s instructions. The library was constructed through sequencing performed using a MiSeq system (Illumina, San Diego, CA, USA), and the paired-end reads (2 × 300 nt) were obtained using a MiSeq Reagent Kit V3 (600-cycle).

### 2.11. Statistical Analysis

All data are presented as means ± standard deviations (SDs). All the statistical analyses were performed using GraphPad Prism 6.0 (GraphPad Software, San Diego, CA, USA). The statistically significant differences were identified using one-way analysis of variance (ANOVA) and validated using a Duncan post hoc test. The significance level was set to *p* < 0.05.

## 3. Results

### 3.1. Effects of Phlorotannin-Rich Extracts from A. nodosum and F. vesiculosus on Body Weight, Food Intake, Food Energy, and Feed Efficiency of Rats with HED-Induced Hyperlipidemia

**Figure 1:**
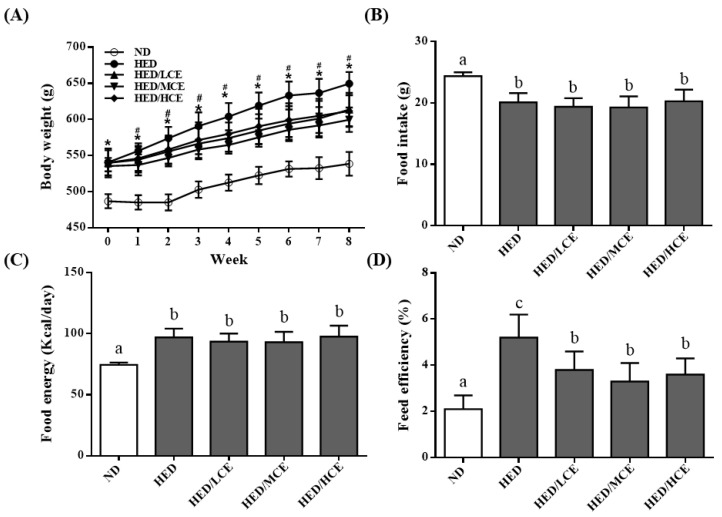
Effects of phlorotannin-rich extracts from *A. nodosum* and *F. vesiculosus* on (**A**) body weight, (**B**) food intake, (**C**) food energy, and (**D**) feed efficiency of rats with high-energy diet–induced hyperlipidemia. ND: normal diet; HED: high-energy diet; LCE: low dosage of crude extract; MCE: medium dose of crude extract; HCE: high dosage of crude extract. Values were presented as means ± SD (n = 10). Data were statistically analyzed using one-way ANOVA followed by a Duncan post hoc test. Different letters and symbols indicate significant differences (*p* < 0.05). * *p* < 0.05 compared with ND; ^#^ *p* < 0.05 compared with HED.

We analyzed the effects of brown algae crude extract on the body weight, food intake, food energy, and feed efficiency of rats with high-energy diet–induced hyperlipidemia. The rats fed an ND and HED gained an average of 53.4 ± 12.6 and 88.3 ± 21.4 g, respectively, by the end of the study (*p* < 0.05). Interestingly, the rats fed an HED containing phlorotannin-rich brown algae crude extract gained significantly less weight than did those who were fed the HED alone, especially after 1 week into the intervention period (*p* < 0.05; Figure 1A). Despite the difference in weight gain between the HED and HED/HCE groups, no significant differences in food intake and food energy were identified (Figure 1B,C). The feed efficiency of the HED group was significantly higher than that of the ND group (Figure 1D). In addition, brown algae crude extract (at all doses) significantly attenuated the increase in feed efficiency caused by the HED.

### 3.2. Effects of Phlorotannins-Rich Extracts from A. nodosum and F. vesiculosus on Fat Pads of Rats with HED-Induced Hyperlipidemia

The average masses of different fat tissues at the end of the study are presented in Table 1. The epididymal, perirenal, and mesenteric fat pads are white adipose tissues (WATs). The average masses of the epididymal, perirenal, and mesenteric fat pads in the HED group were greater than those in the ND group, but those in the HED/LCE, HED/MCE, and HED/HCE groups were lower than those in the HED group. The average body fat percentages of the ND, HED, HED/LCE, HED/MCE, and HED/HCE groups were 3.32% ± 0.77%, 9.24% ± 1.63%, 7.34% ± 1.06%, 7.51% ± 1.12%, 7.33% ± 1.14%, respectively. The average body fat percentage of the rats in the HED group was 178.3% higher than that of the rats in the ND group (*p* < 0.05). The average body fat percentages of the HED/LCE, HED/MCE, and HED/HCE groups were markedly lower than that of the HED group. Furthermore, the brown adipose tissue (BAT)/WAT ratio in the HED group was significantly lower than that in the ND group (*p* < 0.05). Moreover, the HED/LCE, HED/MCE, and HED/HCE group had significantly lower BAT/WAT ratios than did the HED group.

### 3.3. Effects of Phlorotannin-Rich Extracts from A. nodosum and F. vesiculosus on Serum Biochemical Parameters of Rats with HED-Induced Hyperlipidemia

The effects of brown algae crude extract on serum biochemical parameters are presented in Table 2. The HED group had higher blood glucose and lower insulin levels than did the ND group. However, HED/LCE, HED/MCE, and HED/HCE groups had significantly lower glucose levels and higher insulin than did the HED group. The average serum levels of AST and ALT, indicators of hepatic injury, in the HED group (202.3 ± 30.1 and 51.5 ± 15.4 U/L, respectively) were significantly higher than those of the ND group (142.5 ± 23.5 and 43.6 ± 17.1 U/L, respectively; *p* < 0.05). The HED/LCE, HED/MCE, and HED/HCE groups had significantly lower levels of AST and ALT than did the HED group (*p* < 0.05). The average levels of uric acid and creatinine, indicators of nephrotoxicity, did not differ significantly among all groups (*p* > 0.05). In addition, the levels of ketone bodies and electrolytes, including sodium and potassium, in the serum did not differ significantly (*p* > 0.05).

### 3.4. Effects of Phlorotannin-Rich Extracts from A. nodosum and F. vesiculosus on Lipid Indices of Rats with HED-Induced Hyperlipidemia

**Figure 2:**
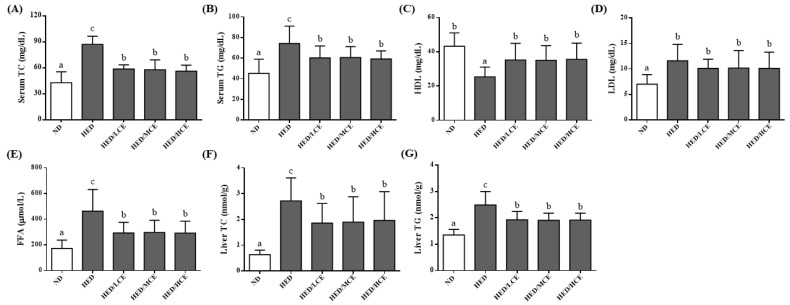
Effects of phlorotannin-rich extracts from *A. nodosum* and *F. vesiculosus* on serum (**A**) TC, (**B**) TG, (**C**) HDL-C, (**D**) LDL-C, (**E**) FFAs, and liver (**F)** TC and (**G**) TG of rats with high-energy diet–induced hyperlipidemia. ND: normal diet; HED: high-energy diet; LCE: low dosage of crude extract; MCE: medium dose of crude extract; HCE: high dosage of crude extract; TC: total cholesterol; TG: triacylglyceride; HDL-C: high-density lipoprotein cholesterol; LDL-C: low-density lipoprotein cholesterol; FFAs: free fatty acids. Values were presented as means ± SD (n = 10). Data were statistically analyzed using one-way ANOVA followed by a Duncan post hoc test. Different letters and symbols indicate significant differences (*p* < 0.05).

To study the effects of brown algae crude extract on lipid metabolism, we assessed the rats’ serum lipid indices, including TC, TG, HDL-C, LDL-C and FFAs levels, and hepatic TC and TG levels (Figure 2). After 12 weeks of HED, the average serum TC, TG, LDL-C and FFAs levels and hepatic TC and TG levels of the HED group were greater than those of the ND group. The HED/LCE, HED/MCE, and HED/HCE groups had lower serum TC, TG, and FFAs levels and hepatic TG level than did the HED group. In addition, the HED group had significantly lower serum levels of HDL-C than did the ND group. The HED/LCE, HED/MCE, and HED/HCE groups had significantly higher serum levels of HDL-C than did the HED group.

### 3.5. Effects of Phlorotannin-Rich Extracts from A. nodosum and F. vesiculosus on Proinflammatory Factors and Antioxidant Enzyme Activities in the Serum of Rats with HED-Induced Hyperlipidemia

**Figure 3:**
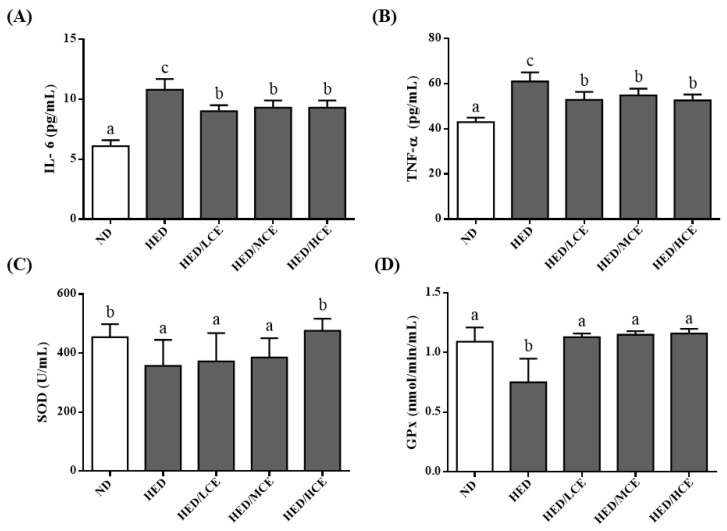
Effects of phlorotannin-rich extracts from *A. nodosum* and *F. vesiculosus* on levels of proinflammatory factors (**A**) IL-6 and (**B**) TNF-α and activities of antioxidant enzymes (**C**) SOD and (**D**) GPx in the serum of rats with high-energy diet–induced hyperlipidemia. ND: normal diet; HED: high-energy diet; LCE: low dosage of crude extract; MCE: medium dose of crude extract; HCE: high dosage of crude extract. Values were presented as means ± SD (n = 10). Data were statistically analyzed using one-way ANOVA followed by a Duncan post hoc test. Different letters and symbols indicate significant differences (*p* < 0.05).

To determine the effects of brown algae crude extract on proinflammatory cytokines and antioxidant enzyme activities, we examined the levels of IL-6 and TNF-α and activities of SOD and GPx in the rats’ serum samples (Figure 3). The HED group had significantly higher IL-6 and TNF-α levels and lower SOD and GPx activities than did the ND group. However, the HED/LCE, HED/MCE, and HED/HCE groups had significantly lower IL-6 and TNF-α levels than did the HED group. In addition, compared with the HED group, the HED/HCE group had significantly higher SOD activity, and HED/LCE, HED/MCE and HED/HCE groups all had significantly higher GPx activity. These results indicated that brown algae crude extract can significantly decrease inflammation and increase antioxidant enzyme activity in serum.

### 3.6. Effects of Phlorotannin-Rich Extracts from A. nodosum and F. vesiculosus on Lipoprotein Lipase Activity and Mitochondrial Function in Rats with HED-Induced Hyperlipidemia

**Figure 4:**
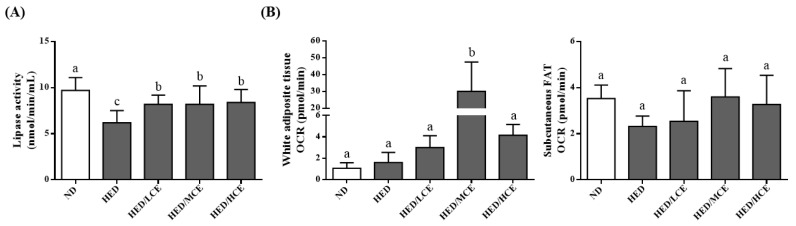
Effect of phlorotannin-rich extracts from *A. nodosum* and *F. vesiculosus* on (**A**) lipoprotein lipase (LPL) activity and (**B**) mitochondrial function of white adipocyte tissue and subcutaneous FAT in rats with high-energy diet–induced hyperlipidemia. ND: normal diet; HED: high-energy diet; LCE: low dosage of crude extract; MCE: medium dose of crude extract; HCE: high dosage of crude extract. Values were presented as means ± SD (n = 10). Data were statistically analyzed using one-way ANOVA followed by a Duncan post hoc test. Different letters and symbols indicate significant differences (*p* < 0.05).

The lipoprotein lipase (LPL) activity of the HED group was much lower than that of the ND group (Figure 4A). The HED/LCE, HED/MCE, and HED/HCE groups had significantly higher LPL activity than did the HED group. In addition, when we assessed mitochondrial function by measuring the rats’ real-time basal OCRs, the subcutaneous fat OCR of the HED group was slightly lower than that of the ND group, and those of the HED/LCE, HED/MCE, and HED/HCE groups were slightly higher than that of the HED groups; however, no significant between-group differences were identified (Figure 4B).

### 3.7. Effects of Phlorotannin-Rich Extracts from A. nodosum and F. vesiculosus on Mean Adipocyte Size of Rats with HED-Induced Hyperlipidemia

**Figure 5:**
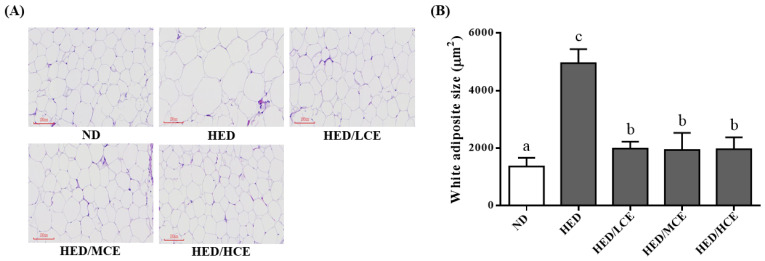
Effect of phlorotannin-rich extracts from *A. nodosum* and *F. vesiculosus* on (**A**) hematoxylin and eosin staining and (**B**) mean adipocyte size of epididymal white adipose tissue in rats with high-energy diet–induced hyperlipidemia. ND: normal diet; HED: high-energy diet; LCE: low dosage of crude extract; MCE: medium dose of crude extract; HCE: high dosage of crude extract. Values were presented as means ± SD (n = 10). Data were statistically analyzed through one-way ANOVA followed by a Duncan post hoc test. Different letters and symbols indicate significant differences (*p* < 0.05).

The HED group had a significantly higher mean adipocyte size than did the ND group (Figure 5). After 8 weeks of treatment with brown algae crude extract, the mean adipocyte sizes of the HED/LCE, HED/MCE, and HED/HCE groups were significantly lower than that of the HED group. These results indicated that brown algae crude extract can significantly ameliorate the adverse adipocyte hypertrophy and the formation of adipose depots in the eWAT.

### 3.8. Effects of Phlorotannin-Rich Extracts from A. nodosum and F. vesiculosus on Gut Microbiota of Rats with HED-Induced Hyperlipidemia

**Figure 6:**
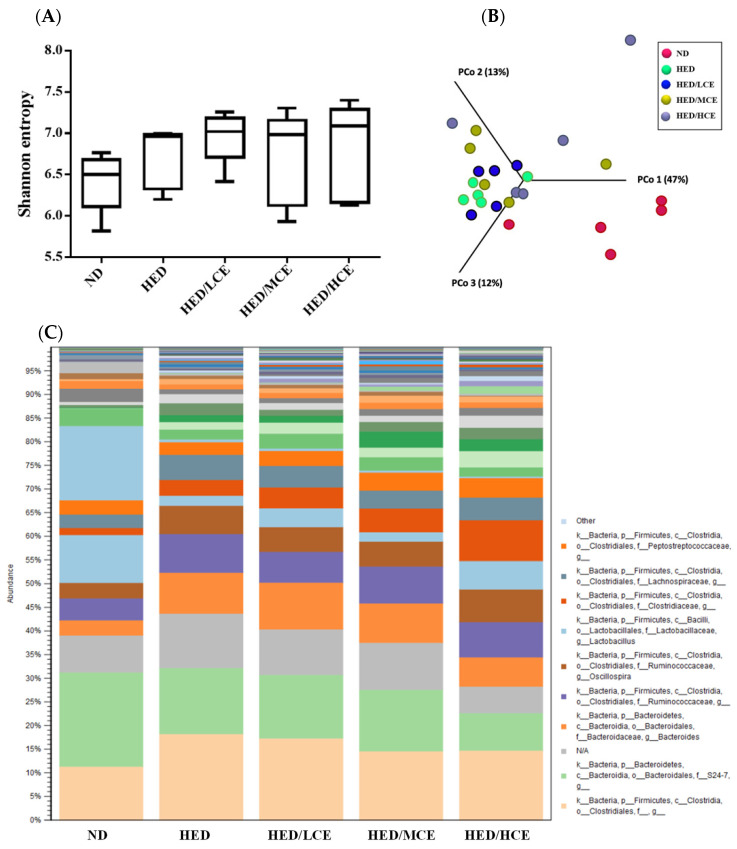
Effect of phlorotannin-rich extracts from *A. nodosum* and *F. vesiculosus* on diversity of gut microbiota in rats with high-energy diet–induced hyperlipidemia. (**A**) Alpha diversity according to Shannon index. (**B**) Beta diversity according to weighted-unifrac principal coordinate analysis plots. (**C**) Relative abundances of microbiota.

To evaluate the effects of brown algae crude extract on gut microbiota composition, we performed 16S rRNA amplicon sequencing on 25 samples (n = 5/group) (Figure 6). We analyzed the gut microbiota composition of the rats in the ND, HED, HED/LCE, HED/MCE, and HED/HCE groups and observed drastic changes in the microbial ecology in the samples of the rats treated with HED for 12 weeks. Moreover, we performed principal coordinate analysis based on weighted-unifrac distances and discovered a high degree of divergence between the rats fed an ND and HED. Unfortunately, the gut microbiota compositions of the HED/LCE, HED/MCE, and HED/HCE groups were similar to that of the HED group. These results suggested that dietary habits play a crucial role in the modulation of gut microbiota composition and that HED may induce dysbacteriosis in the gut. However, the brown algae crude extract did not alleviate or prevent this dysbacteriosis in the present study.

## 4. Discussion

Obesity can lead to excessive TG accumulation in adipose tissue and has adverse health effects, including hyperlipidemia, hypercholesterolemia, hypertension, heart disease, cerebrovascular disease, diabetes, and other chronic diseases [21]. Rats with HED-induced hyperlipidemia have been widely integrated into models used to evaluate methods for preventing or alleviating the symptoms of hyperlipidemia because HED-induced metabolic diseases in rats are similar to metabolic diseases in humans. *A. nodosum* and *F. vesiculosus* contain large amounts of phlorotannins [8]. Researchers have expressed increasing interest in using polyphenols from various traditional herbal medicines to lower blood lipid levels and associated inflammatory responses [22,23,24,25]. In the study, the total phenolic contents in crude extract from *A. nodosum* and *F. vesiculosus* showed the highest amount (39.4 ± 0.3 mg of GAE/g). The present study systematically investigated the effects of phlorotannin-rich brown algae crude extract from *A. nodosum* and *F. vesiculosus* on rats with HED-induced hyperlipidemia.

In this study, the rats in the HED group had a much higher average body weight than did the rats in the ND group. This indicates that the HED group had higher energy intake than did the ND group, and that the HED contributed to the development of obesity. In addition, the average masses of the WATs (epididymal, perirenal, and mesenteric fat pads) in the HED group were higher than those in the ND group. Treating HED rats with LCE, MCE, and HCE for 8 weeks significantly reduced their body weight gain, which may be connected to the lower average mass of the WATs (epididymal, perirenal, and mesenteric fat pads) in the HED/LCE, HED/MCE, and HED/HCE groups. The average size of epididymal fat pads in the HED groups were considerably larger than that of the ND group, but those of HED/LCE, HED/MCE, and HED/HCE groups were smaller than that of the HED group. In addition, the HED group had significantly higher serum levels of glucose, AST, ALT, and FFAs and lower serum levels of insulin than did the ND group. The HED/LCE, HED/MCE, and HED/HCE groups had significantly lower serum levels of glucose, AST, ALT, and FFAs and higher serum levels of insulin than did the HED group. Shepherd and Kahn [26] demonstrated that the plasma FFAs levels of patients with obesity and diabetes are elevated because of the abnormal release of insulin-resistant adipocytes. In patients with insulin resistance, elevated plasma fatty acid concentrations promote hepatic fatty acid synthesis and impair β-oxidation, which may contribute to hepatic steatosis [26]. Paradis et al. [27] discovered that brown seaweed extract of *A. nodosum* and *F. vesiculosus* (at a dosage of 500 mg/day) could modulate insulin homeostasis after ingestion of a carbohydrate-rich meal. Treatment with brown algae crude extract can increase insulin sensitivity and decrease FFAs levels, reducing an individual’s risk of developing hepatic steatosis.

An HED increases serum TC, TG, and LDL-C levels and decreases HDL-C levels, leading to an increased risk of atherosclerosis. Therefore, regulation of blood lipid levels is crucial for preventing atherosclerosis. In our study, we explored the effects of phlorotannins from *A. nodosum* and *F. vesiculosus* on the serum lipid levels of hyperlipidemic rats and discovered that the phlorotannins reduced the rats’ serum TC, TG, and LDL-C levels and increased their HDL-C levels. Nicolucci et al. [28] reported that after patients underwent 6 months of treatment with *A. nodosum* and *F. vesiculosus* extract, their average body weight, waist circumference, fasting blood glucose, HbA1c, systolic and diastolic blood pressure, and LDL-C were decreased and their HDL-C levels was significantly increased. Korukanti et al. [29] reported that feeding rats cafeteria diet for 42 days resulted in a significant increase in the rats’ average body weight and TC, TG, LDL-C and VLDL-C levels and a decrease in their HDL-C levels; however, treatment with *F. vesiculosus* significantly protected against these changes. LPL activity is crucial to the metabolism of lipoproteins and to the regulation of plasma HDL-C levels [30,31,32,33,34,35,36]. Numerous studies have demonstrated that individuals with high plasma LPL activity tend to have high HDL-C levels [30,31,32], which is consistent with the findings of the present study, in which the rats in the HED/LCE, HED/MCE, and HED/HCE groups had high serum LPL activity and high HDL-C levels. Therefore, we proposed that phlorotannin-rich brown algae crude extract from *A. nodosum* and *F. vesiculosus* can decrease serum lipid levels by stimulating serum LPL activity.

In this study, the rats fed an HED had higher serum levels of the inflammatory factors IL-6 and TNF-α and lower activities of the antioxidant enzymes SOD and GPx. However, the rats in the HED/LCE, HED/MCE, and HED/HCE groups had significantly lower IL-6 and TNF-α levels than did those in the HED group. In addition, compared with the HED group, the HED/HCE group had significantly higher SOD activity and the HED/LCE, HED/MCE, and HED/HCE groups had significantly higher GPx activity. Previous studies have demonstrated that phlorotannin-rich extracts from brown algae can effectively control inflammation through various pathways, including inhibition of proinflammatory cytokines (TNF-α, IL-1β, and IL-6) [37]. The extracts from *A. nodosum* and other fucoid species alleviate the effects of oxidative stress by inhibiting reactive oxygen species production, preventing DNA damage, and stimulating glutathione production [38,39,40,41,42,43]. Both in vitro [38] and in vivo [39] experiments have indicated that *A. nodosum* extract has antioxidant and anti-inflammatory properties; therefore, long-term consumption of *A. nodosum* extract polyphenols may have health benefits. Vodouhè et al. [44] reported that consumption of brown algae crude extract of *A. nodosum* and *F. vesiculosus* at a dosage of 500 mg/day for 12 weeks had no effect on body weight or blood glucose; however, early attenuation of the inflammatory response was observed in individuals with overweight/obesity, dysglycemia, and insulin resistance.

## 5. Conclusions

The present study demonstrated that phlorotannin-rich extracts from *A. nodosum* and *F. vesiculosus* may improve the lipid profiles of rats with HED-induced hyperlipidemia by modulating LPL activity, suppressing inflammation, and promoting antioxidant enzyme activity. Therefore, phlorotannin-rich extracts from *A. nodosum* and *F. vesiculosu* may represent a new type of hypolipidemic agent that can help treat hyperlipidemia, insulin resistance, liver steatosis, and associated inflammation.

## Figures and Tables

**Table 1:** Effects of phlorotannin-rich extracts from *A. nodosum* and *F. vesiculosus* on the mass of the epididymal, perirenal, and mesenteric fat pads and total body fat of rats with high-energy diet–induced hyperlipidemia.

	ND	HED	HED/LCE	HED/MCE	HED/HCE
Epididymal fat (g)	5.97 ± 1.47 ^a^	16.82 ± 3.76 ^c^	12.18 ± 2.72 ^b^	12.27 ± 2.36 ^b^	12.26 ± 2.35 ^b^
Perirenal fat (g)	6.81 ± 1.81 ^a^	26.30 ± 4.85 ^c^	20.59 ± 3.59 ^b^	20.54 ± 2.86 ^b^	20.33 ± 3.96 ^b^
Mesenteric fat (g)	5.09 ± 0.99 ^a^	16.41 ± 3.42 ^c^	12.18 ± 2.93 ^b^	12.18 ± 2.45 ^b^	12.17 ± 2.35 ^b^
White adipose tissue (g)	17.87 ± 4.15 ^a^	59.53 ± 10.10 ^c^	44.95 ± 5.73 ^b^	44.99 ± 6.41 ^b^	44.76 ± 6.55 ^b^
Brown adipose tissue (g)	0.47 ± 0.16 ^a^	0.53 ± 0.09 ^a^	0.72 ± 0.16 ^b^	0.72 ± 0.19 ^b^	0.74 ± 0.18 ^b^
BAT/WAT (%)	2.7 ± 1.1 ^a^	0.9 ± 0.2 ^c^	1.6 ± 0.5 ^b^	1.7 ± 0.7 ^b^	1.7 ± 0.4 ^b^
Body fat (%)	3.32 ± 0.77 ^a^	9.24 ± 1.63 ^c^	7.34 ± 1.06 ^b^	7.51 ± 1.12 ^b^	7.33 ± 1.14 ^b^

ND: normal diet; HED: high-energy diet; LCE: low dosage of crude extract; MCE: medium dose of crude extract; HCE: high dosage of crude extract. White adipose tissue (g) = Epididymal fat (g) + Perirenal fat (g) + Mesenteric fat (g). BAT/WAT (%) = [Brown adipose tissue (g)/White adipose tissue (g)] × 100%. Body fat (%) = [White adipose tissue (g)/Body weight (g)] × 100%. Values were presented as means ± SD (n = 10). Data were statistically analyzed using one-way ANOVA followed by a Duncan post hoc test. Different letters and symbols indicate significant differences (*p* < 0.05).

**Table 2:** Effects of phlorotannin-rich extracts from *A. nodosum* and *F. vesiculosus* on serum biochemical parameters of rats with high-energy diet–induced hyperlipidemia.

	ND	HED	HED/LCE	HED/MCE	HED/HCE
FBS (g/dL)	86.1 ± 15.0 ^a^	114.3 ± 5.4 ^b^	80.8 ± 11.1 ^a^	81.2 ± 8.5 ^a^	84.5 ± 14.0 ^a^
Insulin (pg/mL)	144.1 ± 10.9 ^a^	86.0 ± 18.8 ^c^	112.1 ± 10.0 ^b^	113.9 ± 6.8 ^b^	114.5 ± 9.4 ^b^
AST (U/L)	142.5 ± 23.5 ^a^	202.3 ± 30.1 ^c^	172.7 ± 29.8 ^b^	171.6 ± 14.9 ^b^	170.6 ± 23.7 ^b^
ALT(U/L)	43.6 ± 17.1 ^a^	51.5 ± 15.4 ^b^	47.7 ± 11.1 ^a^	46.1 ± 17.0 ^a^	41.4 ± 8.5 ^a^
Uric acid (mg/dL)	1.0 ± 0.4	1.3 ± 0.3	1.1 ± 0.4	1.1 ± 0.3	1.0 ± 0.4
Creatinine (mg/dL)	0.4 ± 0.1	0.4 ± 0.1	0.5 ± 0.1	0.5 ± 0.1	0.5 ± 0.1
Na^+^ (mmol/L)	140.3 ± 3.9	139.8 ± 2.1	138.0 ± 2.2	138.2 ± 3.9	139.3 ± 1.8
K^+^ (mmol/L)	5.0 ± 1.2	5.1 ± 1.1	5.1 ± 1.3	5.1 ± 1.2	5.1 ± 0.7
Ketone body	N.D.	N.D.	N.D.	N.D.	N.D.

ND: normal diet; HED: high-energy diet; LCE: low dosage of crude extract; MCE: medium dose of crude extract; HCE: high dosage of crude extract; FBS: fasting blood sugar; AST: aspartate aminotransferase; ALT: alanine aminotransferase. Values were presented as means ± SD (n = 10). Different letters and symbols indicate significant differences (*p* < 0.05).

## Data Availability

Data is contained within the article.
